# Global Drivers and Tradeoffs of Three Urban Vegetation Ecosystem Services

**DOI:** 10.1371/journal.pone.0113000

**Published:** 2014-11-17

**Authors:** Cynnamon Dobbs, Craig R. Nitschke, Dave Kendal

**Affiliations:** 1 School of Botany, The University of Melbourne, Melbourne, Australia; 2 School of Forest Science and Ecosystem, Melbourne School of Land and Environment, The University of Melbourne, Melbourne, Australia; 3 Australian Research Centre for Urban Ecology, Royal Botanic Gardens Melbourne, c/o School of Botany, The University of Melbourne, Melbourne, Australia; University of Kent, United Kingdom

## Abstract

Our world is increasingly urbanizing which is highlighting that sustainable cities are essential for maintaining human well-being. This research is one of the first attempts to globally synthesize the effects of urbanization on ecosystem services and how these relate to governance, social development and climate. Three urban vegetation ecosystem services (carbon storage, recreation potential and habitat potential) were quantified for a selection of a hundred cities. Estimates of ecosystem services were obtained from the analysis of satellite imagery and the use of well-known carbon and structural habitat models. We found relationships between ecosystem services, social development, climate and governance, however these varied according to the service studied. Recreation potential was positively related to democracy and negatively related to population. Carbon storage was weakly related to temperature and democracy, while habitat potential was negatively related to democracy. We found that cities under 1 million inhabitants tended to have higher levels of recreation potential than larger cities and that democratic countries have higher recreation potential, especially if located in a continental climate. Carbon storage was higher in full democracies, especially in a continental climate, while habitat potential tended to be higher in authoritarian and hybrid regimes. Similar to other regional or city studies we found that the combination of environment conditions, socioeconomics, demographics and politics determines the provision of ecosystem services. Results from this study showed the existence of environmental injustice in the developing world.

## Introduction

Urban areas are dynamic and complex landscapes, where socio-ecological processes can deliver ecosystem services across multiple scales [1]. The ecosystem services concept provides a framework that integrates ecology with socioeconomics, creating a transdisciplinary approach for understanding the benefits that can be delivered by nature and the implications of these benefits on human wellbeing [2], [3]. Population growth, consumption and governance can all influence the provision of ecosystem services which in turn affect human health, livelihood, culture and equity [4]. This concept is particularly relevant in urban systems where natural resources are under enormous pressure and where the demand for ecosystem services is increasing [5].

Cities differ in their governance, infrastructure, economy and social equity [6]. They also vary in their development, with some cities having high rates of urbanization and uncontrolled population growth while other cities are experiencing declines in population. The social, political and biophysical context of the city shapes how socio-ecological interactions affect the provision of environmental benefits [1]. Quantifying how urban ecosystem services are provided under these different socio-political-biophysical conditions provides a useful framework for understanding how socio-political-biophysical factors influence the provision of ecosystem services.

The structure and composition of urban vegetation influences the provision of ecosystem services. A number of regulating services (e.g. maintenance of air quality, climate regulation, maintenance of soil fertility), cultural services (e.g. aesthetics, sense of place and recreation) and supporting services (e.g. habitat for flora and fauna and space for reproduction) are linked to the patterns of urban vegetation [7]–[9]. The distribution of vegetation is a consequence of many factors including topography, climate, transportation infrastructure, plant dispersal mechanisms, real estate markets, planning, cultural practices and social preferences [1], [9]–[12]. Kendal et al. [13] found that temperature influences the composition of cultivated trees in urban areas, while both education and income can influence local vegetation structure and composition [13]–[15]. The relationships between politics and urban vegetation have shown to be characterized by an inequitable distribution, often favouring urban elites over marginalized and deprived groups, either racial or socioeconomic [16]–[19]. However, a global analysis on how governance is related to the provision of ecosystem services is lacking, especially in relation to the national political context. The national political context influences the practice of participatory governance, local-level management and the prioritization of greening policies [20].

We know little about global patterns of ecosystem services, and research at larger scales has generally been restricted to a single country, region or rural landscape [21]. Urban ecosystem services research has focussed mainly on cities in the United States of America [9], [12], [22]–[24] with a few studies in other continents [25]–[29]. These studies mostly focus on the quantification of ecosystem services. We also know little about the tradeoffs and synergies that occur in the provision of ecosystem services [30], particularly in urban landscapes. When assessing services that represent different ecosystem functions, i.e. regulation, supporting and cultural [7], it is necessary to explore their synergies and tradeoffs [31]. Synergies occur when multiple services are simultaneously enhanced, while tradeoffs occur when one service is enhanced at the cost of reducing another [32]. For example, the provision of services such as recreation, spiritual enhancement and psychological benefits typically all increase when the amount and quality of green space available for urban dwellers increases [33]–[35]. In contrast, increasing tree cover in parks leads to increases in carbon storage and habitat provision, but could lead to a reduction in recreational services as the space available for sport fields decreases [36].

To our knowledge, no previous studies have explored the global drivers of urban ecosystem services. This research therefore represents one of the first attempts to quantify global urban ecosystem services and the existence of synergies and tradeoffs and their relation with development, climate and governance. The objectives of the study are 1) to test the effect of socio-political factors on the provision of ecosystem services to explore whether patterns previously found at local-levels scale up globally and 2) to explore whether common biophysical, demography and socioeconomic factors can explain the synergies and tradeoffs in ecosystem services. To achieve our objectives we quantified services that represent different ecosystem functions: carbon storage, recreation potential, and habitat provision. Carbon storage helps mitigate climate change at the global scale by offsetting the urban footprint [37], while at the regional and local scale it contributes to improving air quality [12], [26]. Habitat provision in the urban landscape is strongly linked to biodiversity and to the well-being of urban inhabitants [23], [38]–[43]. In comparison to carbon and biodiversity, recreation potential has a more local effect as it relates to the provision of space for leisure, contemplation and exercising which has been linked to improve public health [44].

## Methods

### Urban vegetation extraction

A sample of one hundred cities was selected to represent a diversity of biophysical, socioeconomic, demographic and cultural factors (Table 1; Figure S1; Table S1). Remotely sensed data were used to provide a standardized method to quantify ecosystem services and look for synergies and tradeoffs across a large number of cities. Cities were selected from a global pool where good quality satellite imagery (Landsat 5 TM) was available during the vegetation-growing season between years 2006 to 2011. Cities from tropical regions in Asia and Africa were not included because of cloud cover over the cities. Landsat images were of high resolution (30 m^2^ multispectral pixels), which allowed for fine scale analysis. Landsat images are widely used in urban landscape studies [45], [46].

**Table 1:** Socioeconomic, political and climatic characteristics for the 100 cities included in this study.

Climate (Köppen classification)	Population	Human Development Index (HDI)	Democracy Index (DI)
Tropical moist (11)	<1 million habitants (11)	Very high HDI (45)	Full democracies (38)
Dry climate (13)	1 to 2 million habitants (32)	High HDI (19)	Flawed democracies (32)
Moist mid latitude with mild winters (60)	2 to 6 million habitants (39)	Medium HDI (22)	Hybrid regimes (14)
Moist mid latitude with cold winters (16)	>6 million habitants (18)	Low HDI (14)	Authoritarian regimes (16)

Identifying city boundaries is a critical step in any analysis of urban landscapes, and one that is notoriously difficult at large scales [47], [48]. The wide range of cities included in the study meant that standardised metadata (e.g. current administrative boundaries) were not available for all cities, therefore a method that could be applied to all cities was required. Following Schneider and Woodcock [47], the limits of a city were defined as the first area where less than 5% impermeable surface was present in a 200 m wide buffer located at the periphery of the urban area. To test the accuracy of this approach, the discrepancy between the administrative and calculated boundary was calculated for 30% of the cities in the study where administrative data was available. Discrepancy varied from a few square meters to 50 km^2^ and was independent of the geographic location of the city, highlighting that that the error associated with our approach was randomly distributed.

The normalized difference vegetation index (NDVI) is an index of living green vegetation [49], and was calculated from the Landsat image of each city, using the red and infrared bands. An unsupervised classification of vegetation and impermeable surface was conducted; however, only vegetation (green cover) was retained for further analysis. To extract the classes representing vegetation the spectral value of 50 vegetation pixels by city were obtained from the NDVI image. The accuracy assessment was obtained using 80 random points within the vegetation class and cross referencing to Google Earth imagery. The Kappa coefficient for the vegetation classification was 0.8, while the user’s accuracy corresponded to 75% and the producer’s accuracy to 85% [50].

### Quantification of ecosystem services

Recreation potential is derived from the vegetated areas that provide space for physical and psychological enjoyment. It includes vegetation in woodlands, grasslands and street trees that occurs in parks, smaller patches of vegetation and/or along streets. The recreation potential service was calculated as the amount of vegetated area per capita [23], [51]. The area of vegetation from the NDVI imagery classification within our calculated urban boundary was divided by the population of the city, obtained from the United Nations global report on human settlements [6]. Due to the lack of data on census limits we assumed that the calculated urban boundary is consistent with the census data limits; while the accuracy of this approach may lead to over or underestimations, the error is randomly distributed and standardised across all cities.

Carbon storage in vegetation was calculated using an existing model based on Landsat derived NDVI [52]. This model was built for urban vegetation and has been validated with field data. The method has been previously used for assessing urban forest carbon offsets and quantifying carbon stock across an urban rural gradient in several cities of the United States [53], [54]. The model is spatially explicit and calculates carbon storage per pixel (30 m^2^) using the function:
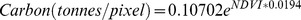



Habitat potential is a function of vegetation structure at the landscape scale. Vegetation cover was obtained from the NDVI analysis. We recognize that different types of vegetation provide different degrees of habitat quality for floral and faunal guilds however this level of detail was not considered in the study. Areas of structural connectivity were identified using a Morphological Spatial Pattern Analysis (MSPA) available in the free software package GUIDOS (http://forest.jrc.ec.europa.eu/download/software/guidos/). MSPA has been used in studies focussed on assessing the connectivity of ecological habitats in both forested and urban landscapes [38], [55], [56]. It uses a land cover map of vegetated/non vegetated areas to classify structural patterns following mathematical morphology methods [57].

The morphological segmentation of binary patterns obtained from an image with vegetated and non-vegetated pixels that produces seven categories according to their size, shape and connectivity.

Our measure of habitat potential is the proportion of area of vegetation larger than 1.44 ha which was classified as ‘core’ habitat [38]. ‘Core’ habitat areas are the pixels in patches where the distance to the non-vegetated area was greater than 60 m (2 Landsat pixels). Our definition of core areas include forest, woodland, shrubland and meadow areas and is consistent with broader ecological theory that shows that larger areas with relatively fewer edges are likely to support a wider of species (such as woodland birds [58]). However, we acknowledge that our measure based on NDVI is not a perfect measure of habitat potential, as it will include large areas of mown turf, which may have low ecological value, and exclude narrow linear corridors that may have high habitat potential. To achieve spatial concordance among the calculated ecosystem services across all cities values were standardised to the mean carbon storage per hectare, mean recreation potential per capita and habitat potential as the proportion of the total urban area covered by ‘core’ patches [59].

### Socioeconomic and climatic factors

To explore the relationship between globally quantified ecosystem services with social and climatic factors we selected three social indicators: total population, the Human Development Index (HDI) and the Democracy Index (DI), and three climatic indicators: mean annual temperature, mean annual precipitation and the annual Heat Moisture Index (HMI) [60].

The Human Development Index (HDI) is a measure of development [61]. It is a composite index that combines indicators of life expectancy, educational attainment and income using a geometric mean [61]. The Democracy Index (DI) is an index that combines five metrics of governance: electoral process and pluralism, civil liberties, the functioning of the government, political participation and political culture [62] (Table S1). Values for mean annual temperature and precipitation were obtained from the World Meteorological Organization (http://www.wmo.int/pages/index_en.html) for the period between 1976 and 2005. The Heat Moisture Index (HMI) was calculated for each city following Wang et al. [60] as a ratio between mean annual temperature and mean annual rainfall. The HMI is a measure of the evaporative demand of the atmosphere and represents the aridity of the environment when the interaction between temperature and precipitation is considered.

### Statistical analysis

The data were tested for normality using the Kolmogorov-Smirnov statistic. Recreation potential was log transformed to meet the analyses’ assumptions of normality. Moran’s I was calculated and used to assess if spatial autocorrelation exists within the analysed data [63].

A principal component analysis (PCA) was applied to the standardized data of each ecosystem service and social-climatic variables included in this study [63]. This multivariate data technique uses orthogonal transformation to group sets of correlated variables into principle components which are sets of linearly uncorrelated variables [64]. A Varimax rotation was used to improve result interpretation. The number of components was selected to provide the most interpretable solution with eigenvalues greater than one.

Following the PCA a Bayesian regression was used to assess the effect size of the socio-climatic variables on each of the ecosystem services considered in the study. This method uses a Markov Chain Monte Carlo technique to fit generalized linear models. Because of the lack of well established relationships between services and indicators, non-informative priors were used. The posterior distribution for each service model was simulated using a Markov chain Monte Carlo method. For each model we simulated 10000 iterations and a burn-in size of 2000, thinning the results by a factor of 1, reaching convergence [63]. For the socio-climatic parameters we report the 2.5% and the 97.5% credible interval of simulated posterior values, which represents the likely range of parameter values. We can be confident of a significant effect where the credible interval does not overlap with zero [65].

Once relations between ecosystem services were established, ANOVAs were used to test for differences between the categories of cities shown in Table 1, using Tukey’s HSD multi-comparison test [63]. These are commonly used categories for classifying cities [6], [61], [62], [66]. Synergies and tradeoffs were identified using pairwise Spearman correlations for all ecosystem services for all the cities together and separated by bio-socio-political relevant factors. Significance of the correlations was assessed at *p*<0.001, *p*<0.05 and *p*<0.01.

## Results

### Quantification of global ecosystem services

There was high variability in green cover. The city with the lowest amount of vegetation cover was Calcutta (0.4%), while Winnipeg had the highest (63.1%). The percent of green cover was normally distributed (D = 0.99, p<0.001), with a mean of 32.6% (±12.1) with the majority of cities having between 20% and 40% green cover. Moran’s I showed no indication of spatial autocorrelation (Z = 0.44, *p* = 0.65). The average recreation potential reached 8.9 m^2^ per capita with the highest value (44 m^2^) for Winnipeg and the minimum value for Istanbul (0.4 m^2^). Carbon storage averaged 39 tonnes per ha, with the lowest value in Khartoum (0.2 tonnes/ha) and the maximum value in Paris (161 tonnes/ha). The average core habitat area provided within city habitat patches was 53%; the highest habitat potential was found for Montreal (98.3%) and the lowest for Bombay (2.4%). Results by city are detailed in Table S2.

### Relationships between ecosystem services and urban climatic and development characteristics

**Figure 1:**
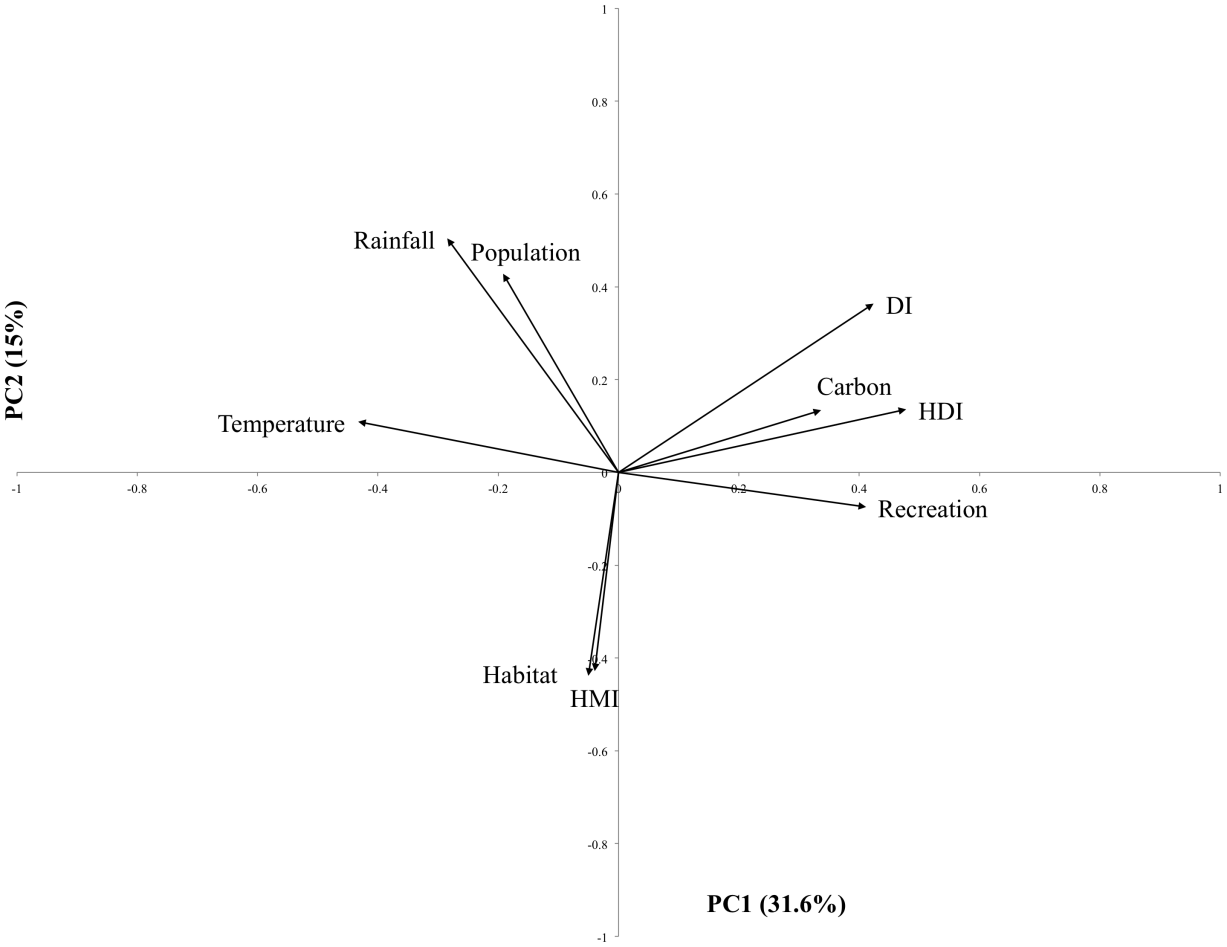
Ordination of the first and second standardized principal component for each ecosystem services and main drivers for 100 cities. The value of PC1 (Principal Component1) and PC2 (Principal Component 2) for the cities was standardised in order to more clearly show their location in the orthogonal space. The length of the arrow is an indication of the strength of the socio-political-climate variables and the ecosystem service in each PC. HMI: Heat Moisture Index, DI: Democracy Index, HDI: Human Development Index.

The PCA was able to detect relationships between urban ecosystem services and development, political and climatic characteristics for the one hundred cities (Figure 1). A two-component solution provided the most interpretable result. Two components explained ∼47% of the variance within the data (Table S3). Principal component 1 explained 31.6% of the variance in the data. The variables that loaded strongly (>0.4) on the first component were recreation potential, temperature, HDI and DI (Figure 1). Habitat provision, rainfall, HMI and population loaded strongly (>0.4) on the second component; this eigenvector explained 14.9% of the variance (Figure 1).

**Figure 2:**
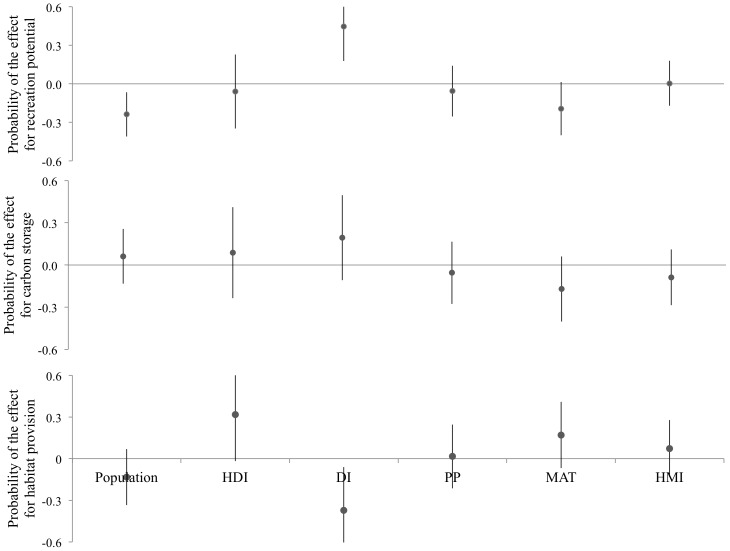
Bayesian models for three ecosystem services. Values overlapping zero imply a consistent effect of the bio-socio-political factor in the probability of having a positive or negative effect in the provision of ecosystem services. HDI: Human Development Index, DI: Democracy Index, PP: Annual precipitation, MAT: Mean Annual Temperature, HMI: Heat Moisture index.

The PCA identified relationships between ecosystem services and some urban characteristics; however, to quantify the effect of these characteristics on services Bayesian regression was used to explore the size and direction of the effect for each urban characteristic (Figure 2; Table S3; Table S4). The association between carbon storage, HDI, DI and temperature, suggests that carbon storage tends to increase in wealthy, educated and democratic cities from cooler climates (Figure 1). The Bayesian analysis resulted in no significant effects detected for carbon storage (Figure 2); however, there were non-significant trends consistent with the PCA results suggesting a weak relationship with temperature and DI.

Recreation potential had a negative relationship with temperature, suggesting that cities from warmer climates tend to have a lower provision of this service. Higher recreation potential tends to occur in more democratic and more highly developed cities. Bayesian analysis showed that DI was the largest (positive) predictor of recreation potential, with population a significant (negative) predictor (Figure 2). There was also a non-significant trend suggesting that temperature may also be negatively related to recreation potential.

Habitat provision was positively related to HMI, suggesting that cooler and wetter cities have a lower provision of this service. Habitat provision had a negative relationship with population, suggesting that increases in population leads to fragmentation of urban vegetation (Figure 1). The Bayesian analysis showed that habitat potential was related (negatively) with DI (Figure 2), suggesting that more democratic cities tend to have lower habitat potential (more fragmented and less connected landscapes). A positive but non-significant trend was also detected between HDI and habitat potential.

The results from the ANOVAs and multiple comparison tests for the provision of ecosystem services by categories of city population, democratic regime and climate are summarised in Table 2. Carbon storage was higher in full than flawed democracies and authoritarian regimes. The analysis identified thresholds where the provision of ecosystem services declines. Cities with less than one million people tended to provide more recreation potential than larger cities, with megacities providing the least amount of recreational space. A higher provision of recreation service occurred in full democracies, and in continental cities over tropical and Mediterranean cities. Interestingly, authoritarian regimes tend to have a higher provision of habitat suggesting a higher degree of connectivity.

**Table 2:** Significant differences (ANOVA) in the provision of ecosystem services using categories from significantly influential urban characteristics.

		Recreation potential (m^2^ per capita)	Carbon storage (kg/ha)	Habitat provision (%)
	*r^2^*	*0.16*	*0.01*	*0.02*
Population	<1 million	16.5^a^ (2.6–44)	39.5^a^ (0.7–81)	45^a^ (5–74)
	1 to 2 million	8.6^b^ (1.7–31)	42.3^a^ (0.7–151)	54^a^ (4–93)
	2 to 6 million	7.5^b^ (0.6–33)	38.4^a^ (0.2–149)	56.2^a^ (4–98)
	>6 million	3.2^b^ (0.4–7.6)	27.7^a^ (5–161)	49.3^a^ (2–93)
	*p-value*	*0.0008*	*ns*	*ns*
Democracy Index	*r^2^*	*0.29*	*0.14*	*0.04*
	Authoritarian	3.4^a^ (0.8–6.5)	21.3^b^ (0.2–59)	63.4^a^ (7–93)
	Hybrid	3.3^a^ (0.4–8)	32^ab^ (3.1–114)	52.6^b^ (4–78)
	Flawed democracy	5.6^a^ (0.7–31)	31.5^b^ (0.7–161)	52.9^b^ (2–90)
	Full democracy	13.9^b^ (1–44)	55.5^a^ (3.4–151)	48.9^b^ (4–78)
	*p-value*	*<0.0001*	*0.002*	*0.01*
Climate	*r^2^*	*0.10*	*0.03*	*0.02*
	Tropical	3.3^a^ (0.6–8)	25.5^a^ (0.7–94.1)	58.2^a^ (2–88)
	Desert	8.5^ab^ (0.6–29)	31.7^a^ (0.2–84)	61.5^a^ (7–90)
	Mediterranean	7.4^a^ (0.4–33)	40.8^a^ (1.9–161)	51.6^a^ (4–93)
	Continental	13.7^b^ (3–44)	46.5^a^ (0.7–103)	49.2^a^ (4–98)
	*p-value*	*0.01*	*ns*	*ns*

Values label with the different letter a imply significant differences among categories of analysis for Tukey’s HSD comparison test.

### Synergies and tradeoffs of urban ecosystem services

There was a synergy between recreation potential and carbon storage across all cities (Table 3), which were positively and moderately correlated (Spearman’s ρ>0.5, *p*<0.001). There were no significant correlations between habitat provision and recreation, or between carbon storage and habitat provision. Within city categories, the synergy between recreation and carbon is consistent for all population sizes with cities over 1 million people showing stronger correlations (Spearman’s ρ≈0.6) than cities under 1 million (Spearman’s ρ≈0.45). This synergy is also consistent for different political regimes except for full democracies. The synergy is strongest in Desert and Mediterranean cities and does not hold for continental and tropical cities. Synergies between recreation potential and habitat provision are only present in cities with under 1 million inhabitants and in democracies. A weak but significant synergy (*p*<0.01) was found in Mediterranean cities between carbon storage and habitat provision (Table 3).

**Table 3:** Spearman correlations between different ecosystem services for all the cities and by categories of population, democracy and climate.

		Recreation vs. Carbon	Recreation vs. Habitat	Carbon vs. Habitat
All cities		0.53***	0.09	0.02
Population	Less 1 million	0.46*	0.47*	0.009
	1 to 2 million	0.64 ***	0.1	0.05
	2 to 6 million	0.59***	0.24	0.11
	more than 6 million	0.62**	−0.12	−0.14
Democracy	Authoritarian regimes	0.47**	0.07	−0.04
	Hybrid regimes	0.66**	−0.11	−0.02
	Flawed Democracy	0.63***	0.25*	0.17
	Full Democracy	0.12	0.36*	0.15
Climate	Tropical	0.18	0.3	−0.19
	Desert	0.64**	0.009	−0.1
	Mediterranean	0.58***	0.12	0.19*
	Continental	0.27	0.24	0.05

Fisher significant test: *p-value<0.01, **p-value<0.05, ***p-value<0.001.

## Discussion

Three ecosystem services were characterized for one hundred cities, revealing that the global distribution of ecosystem services are shaped by both development factors and climate. By integrating NDVI based land cover information with development indicators we demonstrated that anthropogenic variables do influence the provisioning of ecosystem services within urban systems [32], [67], [68]. Population and political factors more directly influenced recreation potential and habitat potential, while carbon storage was influenced by political regime and temperature. Similar to other urban studies, ecosystem services were influenced by both the natural environment (climate) and by demographics, socioeconomics and governance [14], [69]–[71]. The congruence with local and regional studies demonstrates that these aforementioned relationships scale up to the global scale and highlight the need to consider socio-political-environmental dimensions when developing urban areas to achieve sustainable development goals.

Our study found that recreational potential is lower in cities with more than 1 million inhabitants, which is consistent with trends observed in some European cities [51]. Recreation potential also decreased with changes in governance as cities in countries governed by non-democratic regimes had a lower provision of this service. It is well understood that developing countries have social inequalities caused by urbanization [72], and this study highlights the existence of environmental inequalities that may be exacerbate by urbanization. Of the 100 cities studied, only 26% of the cities had more green cover person than World Health Organization (WHO) recommendations of 9 m^2^ per capita [73] and only 12% of the cities met the green space per capita of 20 m^2^ per capita [74], [75]. The only cities that reached the WHO recommendation were mid-size cities (1 to 6 million people) that are predominantly located in North America and South Africa, with the exception of Bismarck, Oklahoma City and Winnipeg all located in North America.

Cities with higher HDI and under democratic regimes were found to provide more green space for their inhabitants, which may be due to the increased demand for environmental quality by residents [76]. This group included cities in Canada, which have strong environmental and urban forestry programs and policies at both the national and local level. These policies promote an increase in urban green space and street tree plantings and the maintenance of conservation areas [77]. At the opposite end of the spectrum are cities with low HDI, where the main policies at the country and local level are mainly related to socioeconomics; in addition, many of these countries have poor institutional capacity and insufficient budgets to deliver environmental policies [78], [79].

Provision of the carbon storage service was found to be strongly influence by climate, though HDI and DI (in interaction with HDI) were also important factors. Cities with the highest provision of this service were mostly from continental biomes (Frankfurt, Paris, Prague), while the lowest provisioning of this service in its majority occurred in cities located in tropical to desert biomes (Ulaanbaatar, Sana’a, Pyonyang). Certain cities however were outliers, interestingly Mendoza, Phoenix and Las Vegas have been able to increase carbon storage despite being located in desert biomes [80], [81]. Humans have greatly increased the number of trees within cities such as Phoenix, U.S.A. and Mendoza, Argentina, despite climatic limitations, thereby increasing the provision of this service. In general, more affluent and democratic cities are more likely to have a greater biomass of vegetation that can sequester more carbon, either by maintaining larger patches of vegetation and, or in the case of many continental cities, larger tree populations in streets.

Habitat potential was mainly related to HDI and governance, which is consistent with other studies (e.g. Schwarz 2010; Huang et al. 2007). Cities governed by flawed democracies, authoritarian and hybrid regimes from low-income countries tend to be more compact, with vegetation restricted to peri-urban areas and consequently have low levels of fragmentation. This might reflect the effect of motorization with developed countries with full democracies as they tend to be sprawling cites characterized by high levels of vehicle ownership and the associated transportation infrastructure required to facilitate commuting by vehicles [71]. In addition, control over land ownership under socialist or communist regimes tend to result in cities that are less fragmented [71], therefore maximizing habitat potential. When a city is governed within a full democratic regime, habitat potential is likely to be reduced. This is particularly apparent in cities where urban development happened between the 18th and 19th century under European colonization and where high rates of sprawling and dispersed urbanization are still prevalent (e.g. U.S.A. and Australia; [71]).

The delivery of ecosystem services varies according to city context. In general, megacities provide low levels of ecosystem services; mid-size cities provided average levels of ecosystem services, while cities with less than 1 million inhabitants typically had higher levels of recreation potential. Fuller and Gaston [51] found the same trend in European cities which highlights that a common signal exists at the global scale. The level of democracy within the country the city is located in affected both recreation potential and carbon storage services positively but had a negative effect on habitat provision. Overall this suggests that inequalities in the provision of ecosystem services exists between less and more developed countries which further supports our thesis that environmental inequalities found within cities maybe relevant at the global scale [17], [83].

The cities that have the highest values for the three services combined in this study were Prague, Paris and Frankfurt, old European cities of very high development level under full democracy. While the lowest values provided for the combined services were Ulaanbaatar, Buenos Aires and Tegucigalpa with medium level of development within flawed and hybrid democracies.

The analysis revealed that synergies between cultural and regulation functions exist as do tradeoffs with some supporting services. Synergies and tradeoffs of this nature have also been found for a variety of other land uses [29], [31]. Relationships between ecosystem services were not linear and varied according to the combination of socioeconomic and political characteristics of the urban ecosystem which is consistent with findings of global coastal ecosystem services [84]. Unsurprisingly, there was a moderately strong synergy between recreation and carbon across most cities as both services are related to the amount of vegetation in cities. However, the synergistic relation between recreation and carbon is very weak for full democracies, which might be due to the existence of parks with relatively few trees, and a relatively larger population of trees located along streets. This is confirmed by the positive relation between recreation and habitat and the weak correlation with habitat and carbon. The relation between recreation and carbon is also weaker in tropical and continental cities. Tropical cities have smaller vegetated areas that are primarily covered by trees, while continental cities have larger vegetated areas cover by fewer trees. There was a substantial variability in the habitat and carbon data however and the power of the analysis may be improved by increasing the sampled size of cities within each climate classification.

The analysis of ecosystem service provision for a hundred cities has its limitations, which need to be acknowledged. City boundaries were difficult to obtain for a wide range of cities; therefore, errors may exist in the estimation of the services. However, for the relationships between democracy, development and climate our methodology allows for a robust assessment. A better estimation of recreation potential could have been obtained if we had further details on the structure and composition of these spaces along with the use of these spaces by people. The resolution of Landsat imagery however was not fine enough to achieve this, and as finer resolution imagery is difficult to source for a large proportion of cities included in this study this level of analysis could not be conducted. The estimation of carbon stored through a model that uses NDVI is commonly used in natural areas and some urban areas [52]–[54], where the outcomes are biased by model performance and the calculation of NDVI; we at least had control over biases and errors in the latter. The habitat potential estimation was not intended to assess the functionality of each vegetation patch and therefore is a coarse metric of habitat. We feel however that the results from this study provide a level of precision that is consistent between cities and our results are consistent with the findings of other urban studies conducted at finer scales.

Further research should include the addition of indicators that can represent cultural background and the legacy of historic development such as the effects of colonization, wars, ethnic diversity, industrialization, planning regulation and infrastructure development, among others [71]. A temporal analysis may also be able to shed light on different urban morphological trajectories and their relationships with ecosystem services. The inclusion of other ecosystem services would reveal more about how the context of the city affects the provision of ecosystem services; however, finding available information for a large range of cities is problematic. Existing standardised global datasets, such as the one developed in this study, are useful to explore the tradeoffs or synergies between services, along with finer scale indicators of biodiversity and other services.

## Conclusion

Cities are areas of human agglomeration that depend on natural resources for the maintenance of human wellbeing. This study has identified that a relationship exists between the bio-socio-political context and the provision of ecosystem services. Cities in countries with democratic systems and more developed economies tend to provide more ecosystem services to their inhabitants; in theory, this should promote improved human wellbeing. This relation becomes more evident for the cultural and provisioning services included in this study, while regulating services such as carbon storage are primarily driven by biophysical conditions followed by social context. The context of the city also influences the synergies and tradeoffs between ecosystem services. This highlights that improvements in economic conditions may not maximise and can hinder the provision of ecosystem services. As a global city scale analysis, this study was able to identify the existence of environmental inequalities according to political, economic and demographic context, which suggests further research should explore these relations within and across cities. Understanding the synergies between services and social and environmental context should ameliorate the development of environmental and social inequalities that are typical of urbanization. The relationship between ecosystem services and bio-socio-political context provides a key understanding of the influential factors that urban planning and policy making impinge upon and thus provide insights for creating liveable, sustainable, and resilient cities globally.

## Supporting Information

Figure S1 **Map of studied cities.**
(TIFF)

Table S1 **List of cities included in this study.** Details on population, Human Development Index and Democracy Index by each city are provided.
(DOCX)

Table S2 **Three urban forest ecosystem services for one hundred cities included in the study.**
(DOCX)

Table S3 **Scores for principal components and their respective eigenvalues.**
(DOCX)

Table S4 **Mean, 2.5% and 97.5% confidence intervals for estimated probabilities of the effect of socioeconomic, political and climate variable using Bayesian regression on each studied ecosystem services.**
(DOCX)
