# Supplementary figures and images for: Global Drivers and Tradeoffs of Three Urban Vegetation Ecosystem Services
Source: PLoS One. 2014 Nov 17;9(11):e113000. doi: 10.1371/journal.pone.0113000 (PMC4234474; doi:10.1371/journal.pone.0113000)

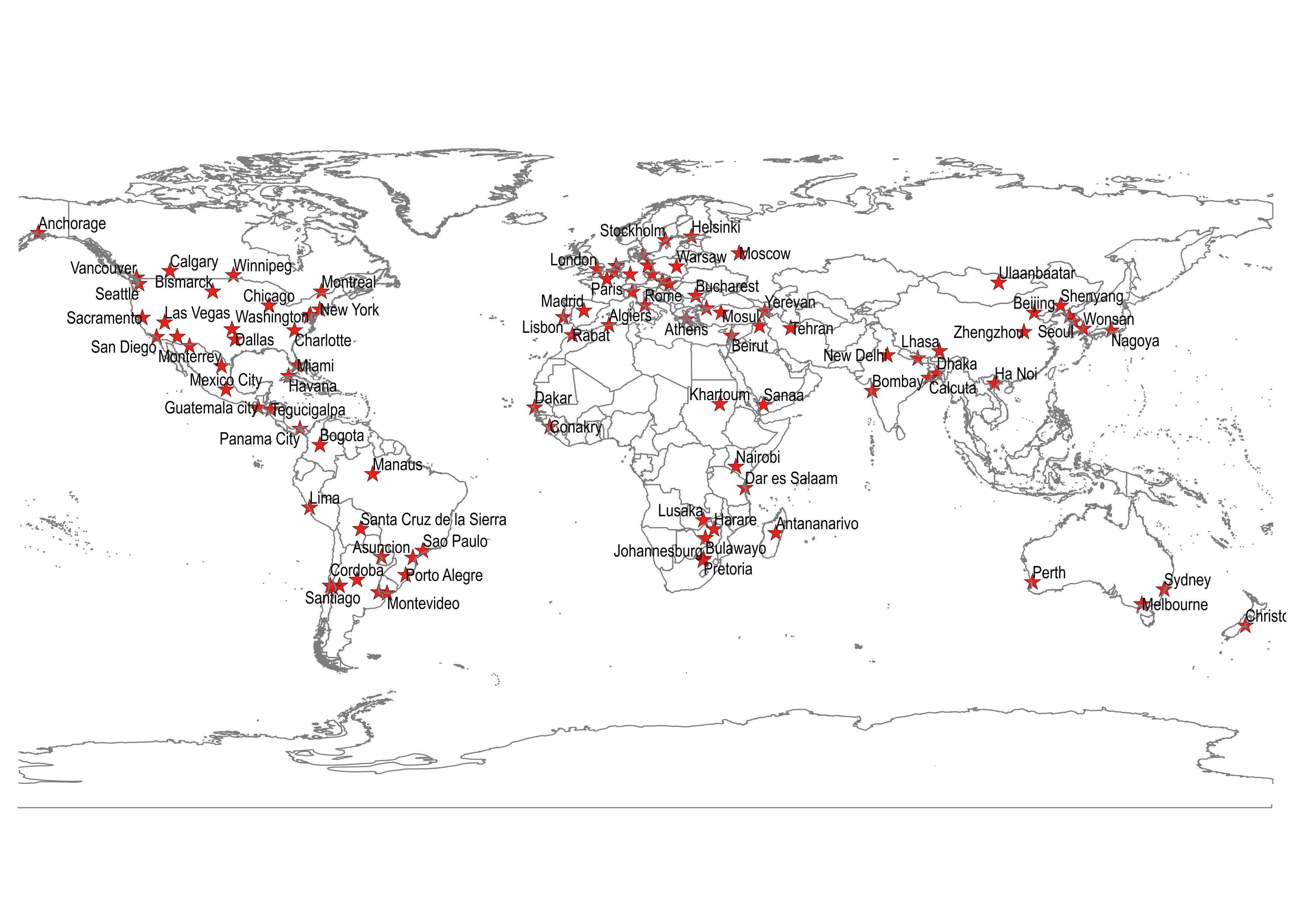

Supplement: Figure S1 — Map of studied cities. (TIFF) [file pone.0113000.s001.tiff]
