# Supplementary material for: Global Drivers and Tradeoffs of Three Urban Vegetation Ecosystem Services
Source: PLoS One. 2014 Nov 17;9(11):e113000. doi: 10.1371/journal.pone.0113000 (PMC4234474; doi:10.1371/journal.pone.0113000)
Supplement: Table S1 — List of cities included in this study. Details on population, Human Development Index and Democracy Index by each city are provided. (DOCX) [file pone.0113000.s002.docx]

| Country | Cities | Climate | Population | HDI | DI |
| --- | --- | --- | --- | --- | --- |
| Algeria | Algiers | Mediterranean | 2 to 6 million | Medium | Authoritarian |
| The Netherlands | Amsterdam | Mediterranean | 1 to 2 million | Very high | Full |
| USA | Anchorage | Continental | less 1 million | Very high | Full |
| Turkey | Ankara | Mediterranean | 2 to 6 million | Medium | Hybrid |
| Madagascar | Antananarivo | Mediterranean | 1 to 2 million | Low | Authoritarian |
| Paraguay | Asuncion | Mediterranean | 2 to 6 million | Medium | Flawed |
| Greece | Athens | Mediterranean | 2 to 6 million | Very high | Flawed |
| USA | Baltimore | Mediterranean | 2 to 6 million | Very high | Full |
| China | Beijing | Continental | > 6 million | Medium | Authoritarian |
| Lebanon | Beirut | Mediterranean | 1 to 2 million | Very high | Hybrid |
| Germany | Berlin | Mediterranean | 2 to 6 million | Very high | Full |
| USA | Bismarck | Continental | less 1 million | Very high | Full |
| Colombia | Bogota | Mediterranean | > 6 million | Medium | Flawed |
| India | Bombay | Tropical | > 6 million | Medium | Flawed |
| Slovakia | Bratislava | Mediterranean | less 1 million | Very high | Flawed |
| Belgium | Brussels | Mediterranean | 1 to 2 million | Very high | Full |
| Romania | Bucharest | Mediterranean | 1 to 2 million | High | Flawed |
| Hungary | Budapest | Mediterranean | 1 to 2 million | Very high | Flawed |
| Argentina | Buenos Aires | Mediterranean | > 6 million | High | Flawed |
| Zimbabwe | Bulawayo | Desert | 1 to 2 million | Medium | Flawed |
| India | Calcutta | Tropical | > 6 million | Medium | Flawed |
| Canada | Calgary | Continental | 1 to 2 million | Very high | Full |
| USA | Charlotte | Mediterranean | 1 to 2 million | Very high | Full |
| USA | Chicago | Continental | > 6 million | Very high | Full |
| New Zealand | Christchurch | Mediterranean | less 1 million | Very high | Full |
| Mexico | Ciudad Juarez | Desert | 1 to 2 million | High | Flawed |
| Madagascar | Conakry | Tropical | 2 to 6 million | Low | Authoritarian |
| Denmark | Copenhagen | Mediterranean | 1 to 2 million | Very high | Full |
| Argentina | Cordoba | Mediterranean | 1 to 2 million | High | Flawed |
| Brazil | Curitiba | Mediterranean | 2 to 6 million | High | Flawed |
| Senegal | Dakar | Desert | 2 to 6 million | Low | Hybrid |
| USA | Dallas | Mediterranean | 2 to 6 million | Very high | Full |
| Tanzania | Dar es Salaam | Tropical | 2 to 6 million | Low | Hybrid |
| Bangladesh | Dhaka | Tropical | > 6 million | Low | Hybrid |
| Germany | Frankfurt | Mediterranean | 2 to 6 million | Very high | Full |
| Guatemala | Guatemala City | Tropical | 1 to 2 million | Medium | Flawed |
| Vietnam | Ha Noi | Mediterranean | 2 to 6 million | Medium | Authoritarian |
| Zimbabwe | Harare | Mediterranean | 1 to 2 million | Low | Authoritarian |
| Cuba | Havana | Tropical | 2 to 6 million | High | Authoritarian |
| Finland | Helsinki | Continental | 1 to 2 million | Very high | Full |
| Turkey | Istanbul | Mediterranean | > 6 million | Medium | Hybrid |
| South Africa | Johannesburg | Mediterranean | 2 to 6 million | Medium | Flawed |
| Nepal | Kathmandu | Mediterranean | 1 to 2 million | Low | Hybrid |
| Sudan | Khartoum | Desert | 2 to 6 million | Low | Authoritarian |
| USA | Las Vegas | Desert | 1 to 2 million | Very high | Full |
| China | Lhasa | Continental | 1 to 2 million | Medium | Authoritarian |
| Peru | Lima | Desert | > 6 million | High | Flawed |
| Portugal | Lisbon | Mediterranean | 2 to 6 million | Very high | Full |
| UK | London | Mediterranean | > 6 million | Very high | Full |
| Zambia | Lusaka | Mediterranean | 1 to 2 million | Low | Hybrid |
| Spain | Madrid | Mediterranean | 2 to 6 million | Very high | Full |
| Brazil | Manaus | Tropical | 1 to 2 million | High | Flawed |
| Australia | Melbourne | Mediterranean | 2 to 6 million | Very high | Full |
| Argentina | Mendoza | Desert | less 1 million | High | Flawed |
| Mexico | Mexico City | Mediterranean | > 6 million | High | Flawed |
| USA | Miami | Tropical | 2 to 6 million | Very high | Full |
| Italy | Milan | Mediterranean | 2 to 6 million | Very high | Flawed |
| Mexico | Monterrey | Desert | 2 to 6 million | High | Flawed |
| Uruguay | Montevideo | Mediterranean | 1 to 2 million | High | Full |
| Canada | Montreal | Continental | 2 to 6 million | Very high | Full |
| Russia | Moscow | Continental | > 6 million | High | Hybrid |
| Iraq | Mosul | Mediterranean | 1 to 2 million | Low | Hybrid |
| Japan | Nagoya | Mediterranean | 2 to 6 million | Very high | Full |
| Kenya | Nairobi | Mediterranean | 2 to 6 million | Low | Hybrid |
| India | New Delhi | Desert | > 6 million | Medium | Flawed |
| USA | New York | Mediterranean | > 6 million | Very high | Full |
| USA | Oklahoma City | Mediterranean | less 1 million | Very high | Full |
| USA | Omaha | Continental | less 1 million | Very high | Full |
| Panama | Panama City | Tropical | 1 to 2 million | High | Flawed |
| France | Paris | Mediterranean | > 6 million | Very high | Flawed |
| Australia | Perth | Mediterranean | 1 to 2 million | Very high | Full |
| USA | Phoenix | Desert | 2 to 6 million | Very high | Full |
| Brazil | Porto Alegre | Mediterranean | 2 to 6 million | High | Flawed |
| Czech Republic | Prague | Mediterranean | 1 to 2 million | Very high | Full |
| South Africa | Pretoria | Mediterranean | 1 to 2 million | Medium | Flawed |
| North Korea | Pyongyang | Mediterranean | 2 to 6 million | Low | Authoritarian |
| Morocco | Rabat | Mediterranean | 1 to 2 million | Medium | Authoritarian |
| Italy | Rome | Mediterranean | 2 to 6 million | Very high | Flawed |
| USA | Sacramento | Mediterranean | 1 to 2 million | Very high | Full |
| USA | San Diego | Mediterranean | 2 to 6 million | Very high | Full |
| Yemen | Sana'a | Desert | 2 to 6 million | Low | Authoritarian |
| Bolivia | Santa Cruz de la Sierra | Tropical | 1 to 2 million | Medium | Hybrid |
| Chile | Santiago | Mediterranean | 2 to 6 million | High | Flawed |
| Brazil | Sao Paulo | Mediterranean | > 6 million | High | Flawed |
| USA | Seattle | Mediterranean | 2 to 6 million | Very high | Full |
| South Korea | Seoul | Continental | > 6 million | Very high | Full |
| China | Shenyang | Continental | 2 to 6 million | Medium | Authoritarian |
| Sweden | Stockholm | Continental | 1 to 2 million | Very high | Full |
| Australia | Sydney | Mediterranean | 2 to 6 million | Very high | Full |
| Honduras | Tegucigalpa | Mediterranean | 1 to 2 million | Medium | Hybrid |
| Iran | Tehran | Mediterranean | > 6 million | High | Authoritarian |
| Mongolia | Ulaanbaatar | Continental | less 1 million | Medium | Flawed |
| Chile | Valparaiso | Mediterranean | less 1 million | High | Flawed |
| Canada | Vancouver | Mediterranean | 2 to 6 million | Very high | Full |
| Poland | Warsaw | Mediterranean | 1 to 2 million | Very high | Flawed |
| USA | Washington | Mediterranean | 2 to 6 million | Very high | Full |
| Canada | Winnipeg | Continental | less 1 million | Very high | Full |
| North Korea | Wonsan | Mediterranean | less 1 million | Low | Authoritarian |
| Armenia | Yerevan | Continental | 1 to 2 million | Medium | Hybrid |
| China | Zhengzhou | Mediterranean | 2 to 6 million | Medium | Authoritarian |
