# Supplementary material for: Global Drivers and Tradeoffs of Three Urban Vegetation Ecosystem Services
Source: PLoS One. 2014 Nov 17;9(11):e113000. doi: 10.1371/journal.pone.0113000 (PMC4234474; doi:10.1371/journal.pone.0113000)
Supplement: Table S2 — Three urban forest ecosystem services for one hundred cities included in the study. (DOCX) [file pone.0113000.s003.docx]

| Cities | Recreation Potential (m^2^ per capita) | Carbon storage (tons/ha) | Habitat Provision (%) |
| --- | --- | --- | --- |
| Algiers | 2.6 | 40.79 | 56.0 |
| Amsterdam | 11.7 | 77.16 | 32.1 |
| Anchorage | 38.7 | 3.42 | 74.6 |
| Ankara | 5.1 | 23.86 | 46.7 |
| Antananarivo | 4.9 | 45.70 | 58.0 |
| Asuncion | 1.6 | 4.08 | 44.6 |
| Athens | 4.2 | 4.46 | 69.0 |
| Baltimore | 5.8 | 47.26 | 67.9 |
| Beijing | 3.4 | 7.37 | 68.4 |
| Beirut | 2.0 | 24.48 | 61.6 |
| Berlin | 6.9 | 31.96 | 4.1 |
| Bismarck | 24.8 | 45.65 | 55.8 |
| Bogota | 0.9 | 5.04 | 58.0 |
| Bombay | 1.7 | 28.13 | 2.4 |
| Bratislava | 5.8 | 12.45 | 58.3 |
| Brussels | 3.6 | 28.68 | 57.4 |
| Bucharest | 4.5 | 50.96 | 59.6 |
| Budapest | 7.3 | 42.05 | 6.0 |
| Buenos Aires | 2.7 | 8.83 | 5.8 |
| Bulawayo | 7.3 | 48.21 | 50.2 |
| Calcuta | 0.7 | 30.86 | 70.2 |
| Calgary | 13.6 | 24.26 | 5.5 |
| Charlotte | 28.8 | 78.35 | 60.4 |
| Chicago | 7.5 | 36.27 | 18.5 |
| Christchurch | 11.3 | 3.72 | 49.8 |
| Ciudad Juarez | 3.3 | 6.92 | 88.2 |
| Conakry | 2.1 | 10.52 | 66.4 |
| Copenhagen | 12.3 | 75.38 | 79.5 |
| Cordoba | 2.2 | 7.12 | 48.7 |
| Curitiba | 6.3 | 63.15 | 59.7 |
| Dakar | 0.6 | 3.10 | 52.9 |
| Dallas | 9.8 | 76.70 | 64.6 |
| Dar es Salaam | 8.2 | 15.40 | 62.8 |
| Dhaka | 0.6 | 12.90 | 53.5 |
| Frankfurt | 5.7 | 136.21 | 75.5 |
| Guatemala City | 3.0 | 17.02 | 61.0 |
| Ha Noi | 1.6 | 3.82 | 70.8 |
| Harare | 5.3 | 28.26 | 71.1 |
| Havana | 6.6 | 46.79 | 88.6 |
| Helsinki | 15.4 | 73.56 | 92.0 |
| Istanbul | 0.4 | 5.61 | 78.6 |
| Johannesburg | 21.5 | 68.57 | 68.9 |
| Kathmandu | 1.7 | 18.61 | 65.2 |
| Khartoum | 2.6 | 0.22 | 83.5 |
| Las Vegas | 29.4 | 20.10 | 71.9 |
| Lhasa | 5.1 | 17.66 | 93.7 |
| Lima | 2.8 | 10.53 | 67.2 |
| Lisbon | 1.0 | 17.41 | 45.8 |
| London | 7.1 | 71.97 | 45.0 |
| Lusaka | 3.8 | 11.43 | 65.9 |
| Madrid | 5.0 | 46.97 | 7.5 |
| Manaus | 3.2 | 0.68 | 79.5 |
| Melbourne | 13.1 | 45.12 | 69.9 |
| Mendoza | 6.7 | 56.61 | 52.9 |
| Mexico City | 2.7 | 8.31 | 57.9 |
| Miami | 3.2 | 94.12 | 41.8 |
| Milan | 14.0 | 58.15 | 53.5 |
| Monterrey | 5.6 | 64.79 | 90.0 |
| Montevideo | 6.9 | 8.17 | 6.5 |
| Montreal | 21.8 | 67.37 | 98.3 |
| Moscow | 4.4 | 103.34 | 68.7 |
| Mosul | 5.6 | 114.46 | 59.4 |
| Nagoya | 9.4 | 56.60 | 64.3 |
| Nairobi | 5.5 | 29.56 | 7.8 |
| New Delhi | 2.7 | 48.50 | 6.6 |
| New York | 7.7 | 33.63 | 45.1 |
| Oklahoma City | 22.6 | 21.13 | 59.1 |
| Omaha | 17.8 | 60.16 | 53.3 |
| Panama City | 4.3 | 13.26 | 59.6 |
| Paris | 5.7 | 161.08 | 67.1 |
| Perth | 6.5 | 12.53 | 47.8 |
| Phoenix | 28.2 | 84.07 | 61.4 |
| Porto Alegre | 2.4 | 8.22 | 56.3 |
| Prague | 22.2 | 151.63 | 66.6 |
| Pretoria | 31.2 | 53.46 | 61.4 |
| Pyongyang | 1.6 | 1.91 | 56.0 |
| Rabat | 4.5 | 18.25 | 7.5 |
| Rome | 4.2 | 28.53 | 49.4 |
| Sacramento | 13.4 | 53.95 | 73.0 |
| San Diego | 13.2 | 50.69 | 6.3 |
| Sana'a | 4.2 | 5.38 | 51.7 |
| Santa Cruz de la Sierra | 2.3 | 10.48 | 54.3 |
| Santiago | 2.5 | 31.33 | 61.6 |
| Sao Paulo | 7.6 | 40.33 | 72.9 |
| Seattle | 4.2 | 149.67 | 53.7 |
| Seoul | 3.0 | 61.97 | 7.4 |
| Shenyang | 3.5 | 24.13 | 60.2 |
| Stockholm | 10.6 | 76.99 | 23.6 |
| Sydney | 3.6 | 7.08 | 34.6 |
| Tegucigalpa | 1.9 | 14.66 | 4.2 |
| Tehran | 0.8 | 16.61 | 93.6 |
| Ulan Bator | 2.6 | 0.77 | 5.0 |
| Valparaiso | 4.0 | 6.50 | 45.9 |
| Vancouver | 33.1 | 89.15 | 58.0 |
| Warsaw | 3.1 | 19.16 | 57.6 |
| Washington | 13.4 | 8.45 | 67.8 |
| Winnipeg | 43.9 | 81.27 | 7.7 |
| Wonsan | 3.4 | 12.60 | 33.0 |
| Yerevan | 4.8 | 60.17 | 54.3 |
| Zhengzhou | 2.0 | 59.98 | 55.8 |
