# Supplementary material for: Global Drivers and Tradeoffs of Three Urban Vegetation Ecosystem Services
Source: PLoS One. 2014 Nov 17;9(11):e113000. doi: 10.1371/journal.pone.0113000 (PMC4234474; doi:10.1371/journal.pone.0113000)
Supplement: Table S3 — Scores for principal components and their respective eigenvalues. (DOCX) [file pone.0113000.s004.docx]

|  | PC1 | PC2 |
| --- | --- | --- |
| Human Development Index | **0.47** | 0.14 |
| Democracy Index | **0.42** | 0.36 |
| Recreation Potential | **0.41** | 0.13 |
| Temperature | **-0.43** | 0.11 |
| Rainfall | -0.29 | **0.51** |
| Population | -0.19 | **0.43** |
| Heat Moisture Index | -0.03 | **-0.43** |
| Habitat Provision | -0.05 | **-0.44** |
| Carbon Storage | 0.34 | -0.07 |
| Eigenvalue | 2.84 | 1.35 |
| Variance (%) | 31.6 | 14.9 |
