# Supplementary material for: Global Drivers and Tradeoffs of Three Urban Vegetation Ecosystem Services
Source: PLoS One. 2014 Nov 17;9(11):e113000. doi: 10.1371/journal.pone.0113000 (PMC4234474; doi:10.1371/journal.pone.0113000)
Supplement: Table S4 — Mean, 2.5% and 97.5% confidence intervals for estimated probabilities of the effect of socioeconomic, political and climate variable using Bayesian regression on each studied ecosystem services. (DOCX) [file pone.0113000.s005.docx]

|  |  | Rainfall | Temperature | HMI | Population | HDI | Democracy Index |
| --- | --- | --- | --- | --- | --- | --- | --- |
| Recreation | 2.5% | -0.25 | -0.39 | -0.17 | -0.41 | -0.34 | 0.18 |
|  | Mean | -0.05 | -0.19 | 0.003 | -0.23 | -0.05 | 0.44 |
|  | 97.5% | 0.14 | 0.01 | 0.18 | -0.06 | 0.22 | 0.71 |
| Carbon Storage | 2.5% | -0.28 | -0.40 | -0.28 | -0.13 | -0.24 | -0.11 |
|  | Mean | -0.05 | -0.17 | -0.08 | 0.06 | 0.08 | 0.19 |
|  | 97.5% | 0.16 | 0.06 | 0.11 | 0.26 | 0.41 | 0.49 |
| Habitat Provision | 2.5% | -0.21 | -0.06 | -0.13 | -0.33 | -0.02 | -0.68 |
|  | Mean | 0.01 | 0.17 | 0.07 | -0.13 | 0.32 | -0.37 |
|  | 97.5% | 0.25 | 0.41 | 0.28 | 0.07 | 0.65 | -0.06 |
